# Optimization of expression and properties of the recombinant acetohydroxyacid synthase of *Thermotoga maritima*

**DOI:** 10.1016/j.dib.2015.09.018

**Published:** 2015-10-03

**Authors:** Mohammad S. Eram, Benozir Sarafuddin, Frank Gong, Kesen Ma

**Affiliations:** Department of Biology, University of Waterloo, Waterloo, Ontario, Canada

**Keywords:** Acetohydroxyacid synthase, Hyperthermophiles, *Thermotoga*, Heat-treatment

## Abstract

The data provide additional support of the characterization of the biophysical and biochemical properties of the enzyme acetohydroxyacid synthase from the hyperthermophilic bacterium *Thermotoga maritima* (Eram et al., 2015) [1]. The genes encoding the enzyme subunits have been cloned and expressed in the mesophilic host *Escherichia coli*. Detailed data include information about the optimization of the expression conditions, biophysical properties of the enzyme and reconstitution of the holoenzyme from individually expressed and purified subunits.

## Specification Table

**Table:** 

Subject area	Biochemistry
More specific subject area	Enzymology
Type of data	Text file and graph
How data was acquired	Data was acquired through experimental procedures
Data format	Analyzed
Experimental factors	Heat-treatment of the cell mass to precipitate host proteins
Experimental features	Expression plasmid construction
Effect of expression temperature on yield
Effect of heat treatment on enzyme purity and activity
Effect of oxygen and temperature on activity of the purified enzyme
Effect of reconstitution on the AHAS activity
Data source location	University of Waterloo, Waterloo, Ontario, Canada
Data accessibility	The data presented in this article is related to [1]

## Value of the data

• The data on expression, assay optimization, and characterization of the AHAS are presented
• The data are valuable in understanding the amino acid biosynthesis pathway in hyper/thermophilic microorganisms
• The procedures used for characterization of hyperthermophilic AHAS is reported.
• The data presented here may serve as an example for the simplicity of the purification of the thermostable proteins from overexpressed in a mesophilic host

## 1 Data, experimental design, materials and methods

### 1.1 Expression of the recombinant proteins

Standard procedures were followed for all DNA manipulation, competent cell preparation and transformation according to the methods described by Sambrook and Russell [2]. DNA was isolated from *Thermotoga maritima* biomass that was grown anaerobically on glucose and yeast extract at 80 °C as described by Huber et al. [3] with modifications as previously described [4]. The medium contained (per liter) KCl, 2 g; MgCl_2_·6H_2_O, 1.42 g; MgSO_4_·7H_2_O, 1.8 g; CaCl_2_·2H_2_O, 0.05 g; NaCl, 20 g; (NH4)_2_CO_3_, 1.14 g; KH_2_PO_4_, 0.05 g; resazurin 0.05 mg, trace minerals as described previously by Balch et al. [5], 10 ml; yeast extract, 2.5 g; and glucose, 4.0 g. Before autoclave the pH of the medium was adjusted to 6.8 using 1 M NaOH.

The amplified coding sequences of the putative catalytic and regulatory subunits were cloned separately into pET30a (+) inducible overexpression vector to produce fusion proteins with N-terminal histidine tags. The recombinant plasmids were transformed into *Escherichia coli* DH5α and subsequently were isolated again and introduced into *E. coli* BL21 (DE3) Rosetta 2 cells. (Table 1).

**Fig. 1:**
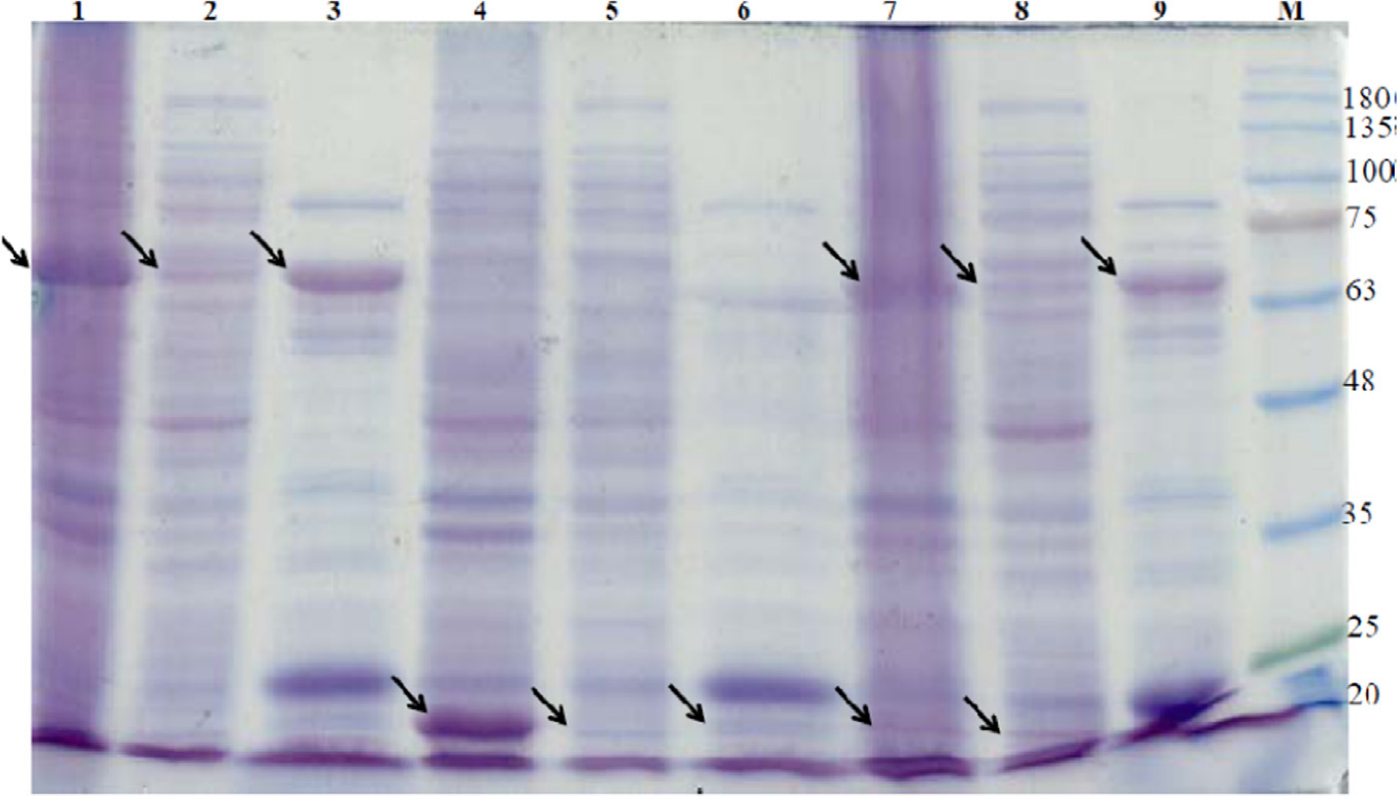
Analysis of over-expression of different clones at 37 °C using SDS-PAGE (12.5%). Lane 1, crude extract of pETTm0548; lane 2, CFE of pETTm0548; lane 3, heat-treated CFE of pETTm0548; lane 4, crude extract of pETTm0549; lane 5, CFE of pETTm0549; lane 6, heat-treated CFE of pETTm0549; lane 7, crude extract of pETTm0548/9; lane 8, CFE of pETTm0548/9; lane 9, heat-treated CFE of pETTm0548/9; M: BLUeye pre-stained Protein Ladder (Froggibio, ON, Canada), the arrows indicate the position of the recombinant protein band; approximately 15 µg protein was loaded per lane.

The clone pETTm0548 produced reasonable amounts of soluble recombinant large (catalytic) subunit (Fig. 1, lanes 1–3); the regulatory (small) subunit recombinant protein showed poor solubility and mainly was expressed as aggregated insoluble protein (Fig. 1, lanes 4–6). In case of the clone pETTm0548/9 the large subunit was expressed in the soluble fractions. The SDS-PAGE data suggest very low yield for the regulatory (small) subunit, which was also very small as indicated by a weak protein band in SDS-PAGE of both soluble and insoluble fractions (Fig. 1, lanes 7–9), suggesting the possible proteolysis or incomplete translation.

### 1.2 Effect of growth temperature on activity

**Fig. 2:**
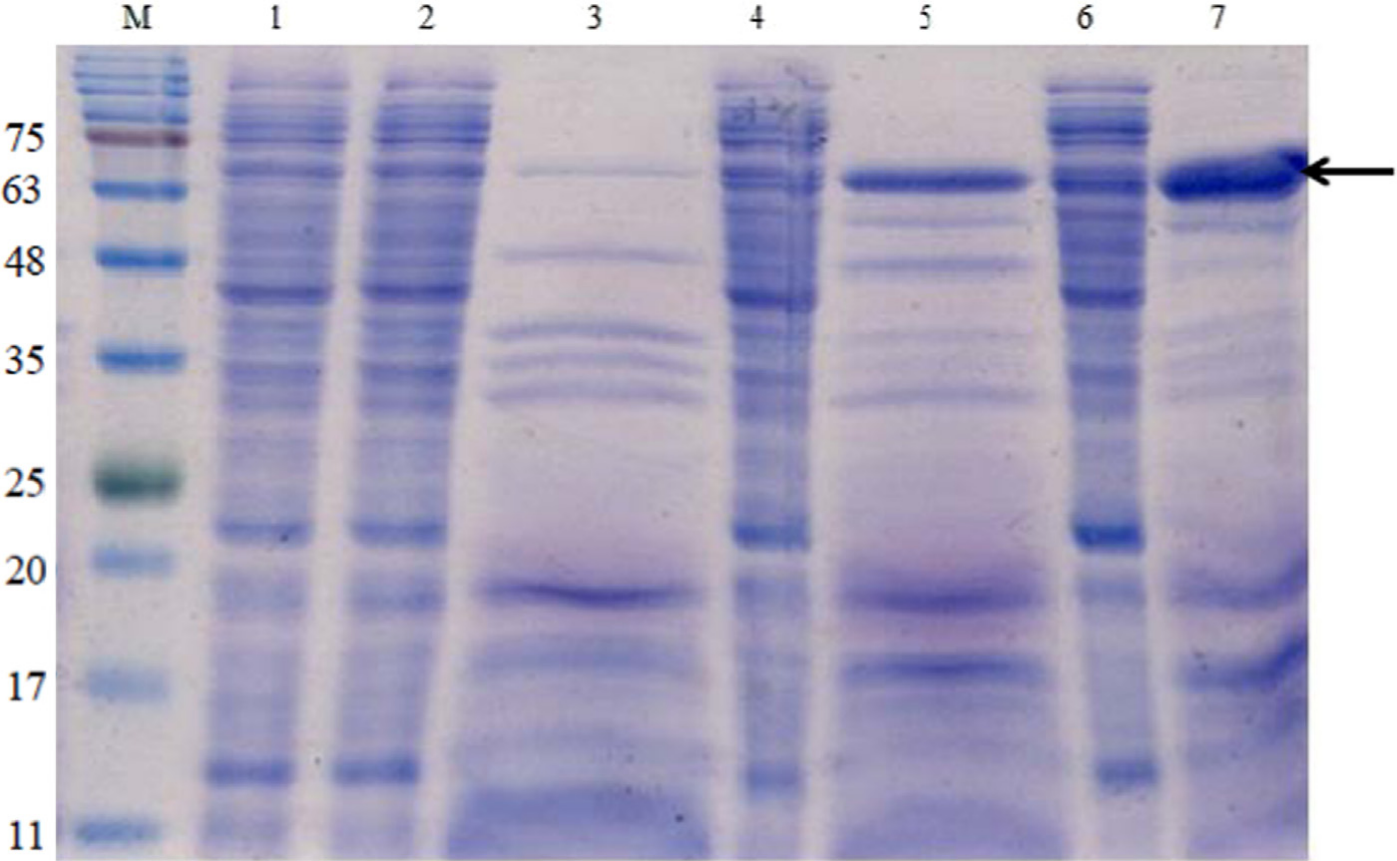
Analysis of the effect of temperatures on expression of pETTm0548 (recombinant catalytic subunit of TmAHAS) using SDS-PAGE (15%). Lane 1, CFE, 18 °C; lane 2, CFE 24 °C; lane 3, heat-treated CFE, 24 °C; lane 4, CFE 30 °C; lane 5, heat-treated CFE, 30 °C; lane 6, CFE, 37 °C; heat-treated CFE 37 °C; M: BLUeye pre-stained protein ladder (Froggibio, ON, Canada), the arrows indicate the position of the recombinant protein band (calculated molecular weight 65.5 kDa); 40 µg of the protein loaded per lane.

**Fig. 3:**
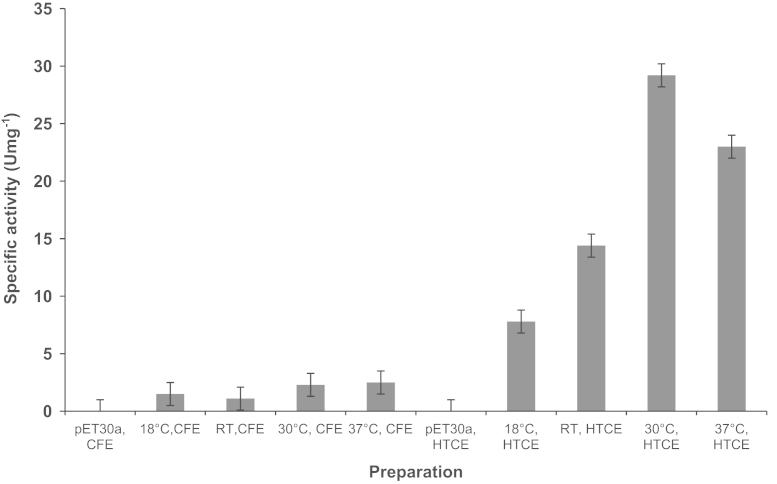
Effect of the expression temperature on AHAS activity of pETTM0548. RT, room temperature (24 °C); CE, crude extract; CFE, cell-free extract; HTCE, heat-treated (80 °C, 1 hr) crude extract.

**Fig. 4:**
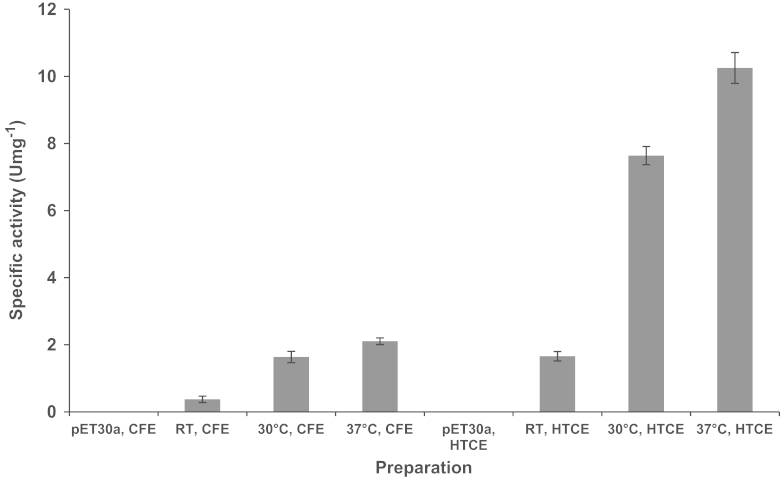
Effect of the expression temperature on AHAS activity of pETTm0548/9. RT, room temperature (24 °C); CE, crude extract; CFE, cell-free extract; HTCE, heat-treated (80 °C, 1 h) crude extract.

Effect of different temperatures on the expression of AHAS clones was tested by incubation of the cultures at 18 °C, 24 °C, 30 °C, and 37 °C following growth at 37 °C and induction. The expression levels were compared using SDS-PAGE analysis (Fig. 2) and enzyme activity assays in the case of the clone expressing the catalytic subunit (Fig. 3) and the clone expressing both subunits together (Fig. 4). The highest yield of soluble recombinant proteins were expressed when the cultures were incubated at 30 or 37 °C following induction.

### 1.3 Choosing the heat-treatment temperature

Heat-induced precipitation (heat-treatment) is widely used for purification of the recombinant thermostable proteins expressed in mesophilic hosts. The optimal temperature for heat-induced precipitation was selected based on the literature data on expression of various hyperthermophilic proteins in *E. coli*. The results of the survey indicated that the heat-precipitation step was mostly successful at the temperature close to the optimal growth temperature of the native organism (Table 2).

### 1.4 Effect of heat-treatment on the activity

**Fig. 5:**
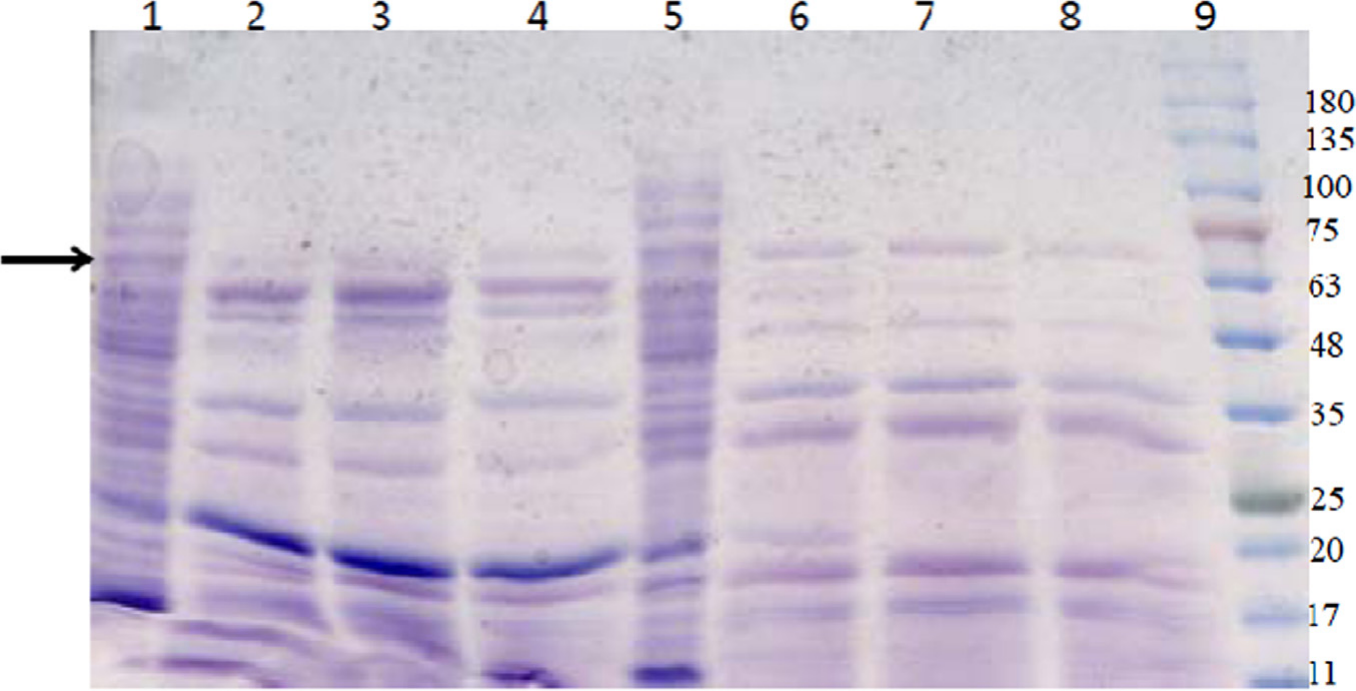
Analysis of the effect of heat-treatment on purification of pETTm0548 (recombinant catalytic subunit of TmAHAS) using SDS-PAGE (12.5%). Lane 1, CFE with no heat-treatment; lane 2, CFE, 30 min at 70 °C; lane 3, CFE, 60 min at 70 °C; lane 4, CFE, 90 min at 70 °C; lane 5, CFE with no heat-treatment; lane 6, CFE, 30 min at 80 °C; lane 7, CFE, 60 min at 80 °C; lane 8, CFE, 90 min at 80 °C; M: BLUeye pre-stained protein ladder (Froggibio, ON, Canada), the black arrow indicate the position of the recombinant protein band (calculated molecular weight 65.5 kDa); approximately 30 µg of the protein loaded per lane.

**Fig. 6:**
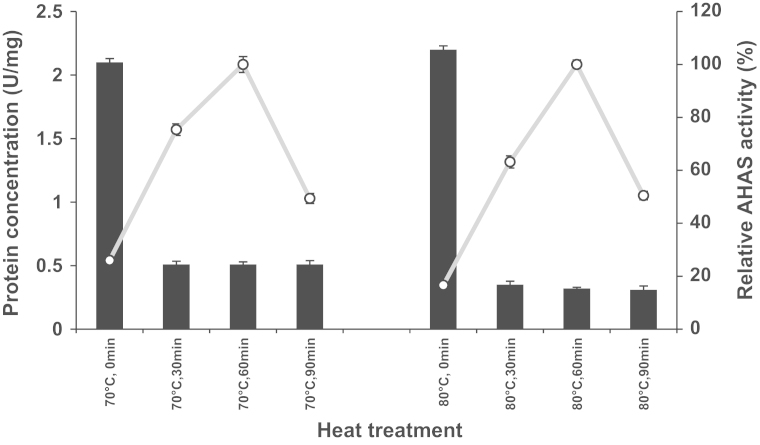
Effect of heat-treatment of crude cell extract of *T. maritima* on AHAS activity. The CFEs were heat-treated at either 70 °C or 80 °C and data were collected by assaying the AHAS activity at different time points. The relative activities were calculated compared to the sample heat treated for 1 h at each temperature. A relative activity of 100% was considered as the highest specific activities measured at each temperature after 1 h of heat-treatment (8.0 U/mg and 8.7 U/mg at 70 and 80 °C). Vertical columns indicate the protein concentration and scatter plots are the relative AHAS activity.

The effect of heating temperatures on the yield of the soluble protein and the corresponding enzyme activity of TmAHAS was tested using crude cell extracts, which were incubated anaerobically at 70 °C and 80 °C, respectively. Samples that were taken at different time intervals were analyzed using SDS-PAGE and enzyme activity assays. The heat-treatment of TmAHAS (catalytic subunit) at 70 °C and 80 °C showed increased purity of the prepared protein over time (Fig. 5). The highest AHAS activity was achieved after heat-treatment at 80 °C for 60 min (Fig. 6). Interestingly, the incubation of the cell crude extracts at any of the two temperatures (70 and 80 °C) resulted in an increased AHAS activity with the highest activity found after 1 h of incubation at each temperature, but extended incubation caused enzyme inactivation (Fig. 6), indicating its thermal stability.

### 1.5 Oxygen sensitivity and thermal stability of the AHAS

**Fig. 7:**
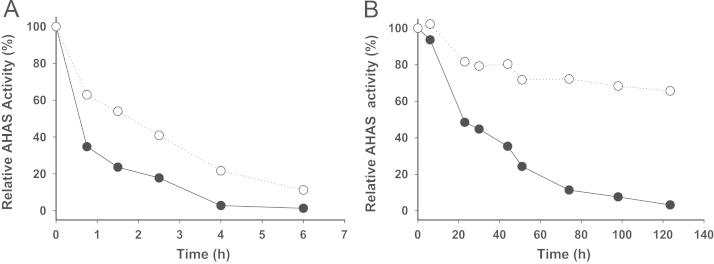
Oxygen sensitivity and thermal stability of TmAHAS activity. (A) The Oxygen sensitivity was determined with the relative activity of 100% considered as the highest activity at time zero without exposure to air (166 U/mg). The filled circles indicate the exposed sample and open circles indicate the un-exposed sample. (B) The thermal stability was determined at 80 °C compared to the enzyme stored at 4 °C as a control. The relative activities of 100% equal to highest measured specific activity at time zero with no heat-treatment (195 U/mg). Filled circles indicate the enzymes incubated at 80 °C and the open circles indicate the enzymes stored at 4 °C.

The data on oxygen sensitivity and thermal stability of TmAHAS was collected by exposing aliquots to ambient atmosphere (at 4 °C) or heat (80 °C) and comparing the activities with unexposed samples at different time intervals (Fig. 7A and B). For anaerobic conditions all of the buffers and reagents were degassed in containers sealed with red rubber sleeved stoppers. The stoppers were punctured with needles to allow the alternate exposure to vacuum and nitrogen (N_2_) using a manifold. The nitrogen gas (Praxair, ON, Canada) was deoxygenated by passing through a heated column containing a BASF catalyst (BASF, NJ, USA). Assay and purification buffers were degassed in magnetically stirred flasks for 30 min; then three cycles of flushing/evacuation (3 min each) were applied. Then a second needle was inserted to flush out more N_2_ to ensure oxygen-free head space in the container (even if there is residual O_2_ contamination in the manifold system). The containers were kept under nitrogen pressure.

### 1.6 Effect of reconstitution on AHAS activity

**Fig. 8:**
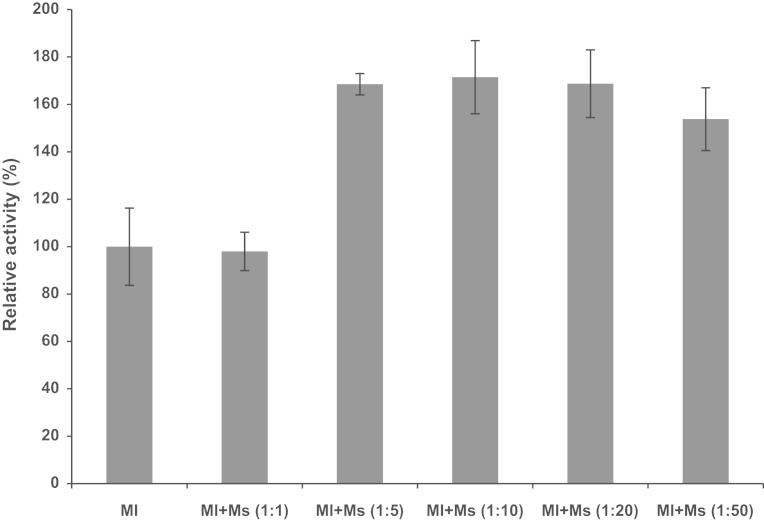
Effect of reconstitution on AHAS activity. Ml, the purified catalytic subunit; Ms, the purified regulatory subunit.

The data indicate the effect of the enzyme reconstitution on AHAS activity of the recombinant AHAS. The purified catalytic subunit (50 pmol) was mixed with the purified regulatory subunit (50, 100, 250, and 500 pmol) in assay mixture. The specific activity of 100% was considered for catalytic subunit alone and was corresponding to 145 U/mg (Fig. 8).

### 1.7 Gel-filtration chromatography of AHAS subunits

**Fig. 9:**
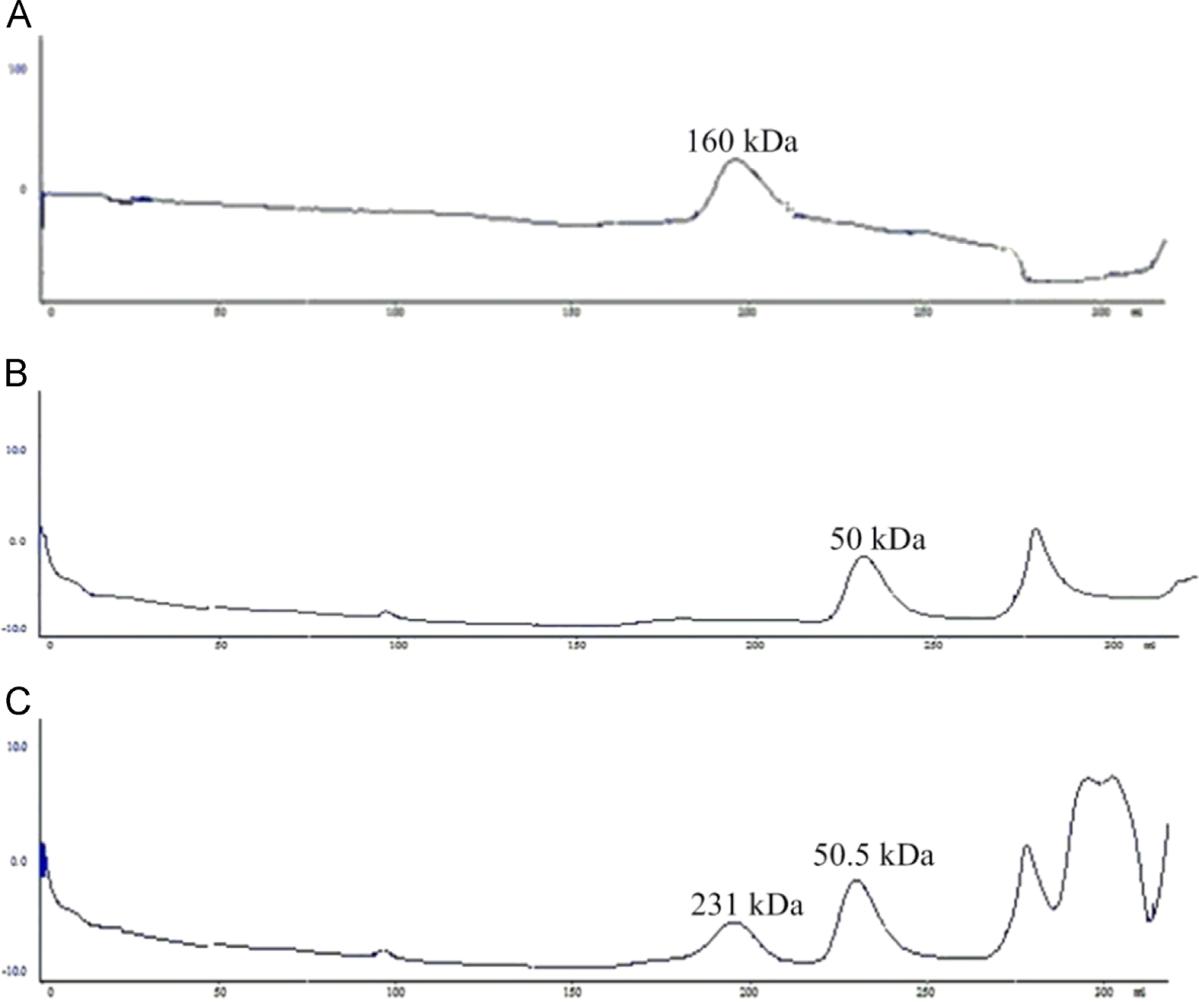
Gel-filtration chromatography of AHAS subunits. Different preparations were loaded on gel-filtration column: (A) catalytic subunit, (B) regulatory subunit, and (C) reconstituted holoenzyme. The numbers indicate the apparent molecular masses of the proteins eluted in each peak. For reconstitution experiment a molar ratio of 1:10 of catalytic subunit to regulatory subunit was mixed together and incubated at room temperature for 1 h. The mixture then was loaded on the size-exclusion column to determine the oligomeric state.

Data on the reconstitution of the large and small subunits were collected by running the catalytic and regulatory subunit individually (Fig. 9A and B) and after mixing (Fig. 9C). The molecular weights of the protein species were determined by loading a size-exclusion chromatography column (2.6 cm×60 cm) of HiLoad Superdex-200 (GE healthcare, QC, Canada) at a flow rate of 2 ml min^−1^. An AKTA FPLC system (GE Healthcare, QC, Canada) was used for running the column pre-equilibrated with 50 mM Tris, 5% glycerol, 100 mM KCl, pH 7.8. The following standards were used: blue dextran (2,000,000 Da), thyroglobulin (669,000 Da), ferritin (440,000 Da), catalase (232,000 Da), aldolase (158,000 Da), bovine serum albumin (67,000 Da), ovalbumin (43,000), chymotrypsinogen A (25,000) and ribonuclease A (13,700) (Pharmacia, NJ, USA).

## Figures and Tables

**Table 1:** Homologs of *ilv* operon in *Thermotogales* and *Thermococcales*^a^.

**Organism**	**AHAS catalytic subunit**^b^**(locus Tag)**	**AHAS regulatory subunit**^b^**(locus Tag)**	**Operon**^c^
*Thermotoga maritima*	TM0548	TM0549	BCAA
*Thermotoga lettingae*	–	–	–
*Thermotoga thermarum*	Theth_0200	Theth_0199	BCAA
*Thermotoga neapolitana*	CTN_0120	CTN_0119	BCAA
*Thermotoga naphthophila*	Tnap_0328	Tnap_0329	BCAA
*Thermotoga petrophila*	Tpet_0372	Tpet_0371	BCAA
*Thermotoga* sp. strain RQ2	TRQ2_0389	TRQ2_0388	BCAA
Thermotogales bacterium MesG1.Ag.4.2^d^	–	–	–
*Thermosipho melanesiensis*	–	–	–
*Thermosipho africanus*	–	–	–
*Petrotoga mobilis*	Pmob_1592	Pmob_1591	BCAA
*Petrotoga miotherma*	–	–	–
*Fervidobacterium nodosum*	–	–	–
*Fervidobacterium pennivorans*	–	–	–
*Kosmotoga olearia*	–	–	–
*Marinitoga camini*	–	–	–
*Marinitoga piezophila*	–	–	–

a The genome sequences were searched by annotation as well as by homology against the protein sequence of the closest known AHAS gene (*T*. *maritima*).

b Alphanumeric codes indicate the locus tag of the gene in the corresponding genome; –, not present.

c The presence of a complete set of *ilv* genes was checked.

d This strain was recently suggested to be named “*Mesotoga prima*” and to be the first member of a new sub-group of mesophilic Thermotogales [6], [7].

**Table 2:** Survey of heat-precipitation temperatures for some recombinant hyperthermophilic proteins expressed in *E. coli*.

**The recombinant protein**	**Native organism**	**Heat-precipitation**	**T**_**opt**_^a^**(**°C**)**	**Reference**
Glutaredoxin-like protein	*Pyrococcus furiosus*	65 °C for 10 min	100	[8]
The HU protein	*Thermotoga maritima*	80 °C for 20 min	80	[9]
Phosphoglycerate kinase	*Thermotoga maritima*	60 min at 80 °C	80	[10]
ADP-dependent phosphofructokinase	*Pyrococcus furiosus*	30 min at 80 °C	100	[11]
Chemotaxis protein	*Thermotoga maritima*	80 °C for 10 min	80	[12]
Maltose-binding protein	*Thermotoga maritima*	75 °C for 30 min	80	[13]
Carboxylesterase	*Sulfolobus sulafataricus*	75 °C for 30 min	80	[14]
Glyceraldehyde-3-phosphate dehydrogenase	*Pyrococcus woesei*	90 °C for 30 min	100–103	[15]
Glyceraldehyde-3-phosphatdee hydrogenase	*Thermotoga maritima*	Inactive protein^b^	80	[16]
Xylose isomerase	*Thermotoga maritima*	90 °C for 2.5 h	80	[17]
l-arabinose isomerase	*Thermotoga neapolitana*	85 °C for 15 min	80	[18]
Alcohol dehydrogenase (*adhC*)	*Pyrococcus furiosus*	80 °C for 30 min	100	[19]
*a*-l-arabinofuranosidase	*Thermotoga maritima*	80 °C for 30 min	80	[20]
6-phosphogluconate dehydrogenase	*Thermotoga maritima*	90 °C for 30 min	80	[21]

a Optimum growth temperature of the native hyperthermophilic organism.

b The heat-treated purified recombinant protein was inactive.
